# EP22 A manifestation of severe sarcoid arthropathy in the distal interphalangeal joint

**DOI:** 10.1093/rap/rkaa052.021

**Published:** 2020-11-03

**Authors:** Rajiv Ark

**Affiliations:** Royal Lancaster Infirmary, Lancaster, United Kingdom

## Abstract

**Case report - Introduction:**

In 2011 a gentleman in his 50s presented with nasal blockage and bloody discharge. He was diagnosed with sarcoidosis and after 9 years of failed strategies to control his disease, he developed dactylitis. X-ray of the hands showed severe arthropathy in the distal interphalangeal joints. This case demonstrates an uncommon extrapulmonary manifestation of sarcoidosis. Although most of his follow up was with a respiratory clinic, his main symptoms were not due to interstitial lung disease, highlighting the importance of a multidisciplinary approach. To reduce the need for steroids, several DMARDs were tried illustrating that there are limited treatment options.

**Case report - Case description:**

This gentleman presented in June 2011 with left epiphora, bloody nasal discharge and fatigue. He had no family history of sarcoidosis and was of Caucasian ethnicity. He was referred by his GP to Ophthalmology and ENT. Septoplasty showed a 95% blockage at the lacrimal sac.

A biopsy was performed, and histology showed a nasal sarcoid granuloma. He was referred to the respiratory team who requested a high-resolution CT scan showing sizeable lymph nodes. One inguinal node was biopsied confirming sarcoid granulomas before starting treatment. Calcium was briefly raised, and serum ACE was initially 123.

He was started on 40mg of prednisolone for 6 weeks, which was tapered to 20/25mg on alternating days. There was a recurrence of his nasal discharge; steroids were increased again but he developed symptoms of muscle weakness from long term steroid use. He was referred to an interstitial lung disease clinic at a tertiary centre where he was investigated for cardiac sarcoidosis with MRI due to ventricular ectopics. Hydroxychloroquine was started to reduce the steroid use however he developed symptoms of tinnitus, so it was stopped. Methotrexate, Azathioprine and Leflunomide were all trialled to however they did not have any impact on controlling his disease. His Prednisolone was slowly reduced by 1mg a month. When he had recurrence of his symptoms, he was given IV methylprednisolone.

Nine years after his first presentation he presented with stiffness of the right thumb base. This progressed to dactylitis and slight fixed flexion deformity of right index finger and left little finger.

An x-ray of his hands showed disease in the distal interphalangeal joints bilaterally with severe changes in the left little finger. The effects of long-term steroids led him to request a letter to support early retirement.

**Case report - Discussion:**

The main rationale for changing treatment options was to reduce the prednisolone dose. Steroids were the only treatment option that showed evidence of controlling his disease when the dose was between 25mg and 40mg a day. Each of the DMARDs that were trialled had a different side effect profile and did not show any evidence of suppressing disease as symptoms recurred. Dose changes later in treatment fluctuated, reflecting a balancing act between disease recurrence and side effects of long-term steroids.

There are many extra pulmonary manifestations of sarcoidosis that were investigated in this case. The first being the nasal granuloma, which can occur in sarcoid patients with symptoms of epistaxis, crusting, congestion, and pain.

There were granulomatous changes seen in the hila as well as other lymph nodes such as the inguinal region; inguinal lymphadenopathy can lead to pain in the groin area.

In addition to this it was important to exclude uveitis with ophthalmology review as he had symptoms of epiphora. Uveitis can be diagnosed in ophthalmological assessment of sarcoid patients in the absence of ocular complaints.

Cardiac sarcoidosis was excluded with an MRI at a specialist heart and lung centre due to ventricular ectopics. Cardiac sarcoidosis can lead to heart block, arrhythmias, and congestive cardiac failure.

Finally, he developed sarcoid arthropathy, review of his radiological images over time showed extensive damage to the joints of the hand.

This gentleman had poor outcomes due to limited treatment options for his disease. Being restricted to long term steroid as the mainstay of treatment led to early retirement due to fatigue and muscle weakness. Conversely, under dosing steroids led to recurrence in symptoms. His disease is still not controlled as shown by an evolving sarcoid arthropathy.

**Case report - Key learning points:**

An illustration of sarcoid arthropathy is also shown in this case. Sarcoid arthropathy is an uncommon manifestation of the disease primarily affecting joints in the hands and feet. In this case the distal interphalangeal joints and proximal interphalangeal joints were affected. The first symptom of arthropathy was stiffness of the base of the right thumb in 2017, this could fit with an osteoarthritic picture and could be mistaken for it in undiagnosed sarcoidosis. The most severe disease was in the DIP of the left little finger, which is not commonly affected. An oligoarthritic pattern with involvement of the ankle is seen more often.

This is also an unusual case of sarcoidosis as there was no family history of the disease and his ethnicity did not predispose him to the condition. He also had a few uncommon extra pulmonary manifestations of sarcoidosis.

The importance of a multidisciplinary approach in managing sarcoidosis was demonstrated in this case. Most of his follow up was with a respiratory clinic. However, respiratory symptoms were not the main issue during the patient journey; early ENT and rheumatology input was significant in managing his disease. Although pulmonary lymph nodes were enlarged, they did not affect his lung function.

